# Correction: Bian et al. Preparation of Sr_2_CeZrO_6_ Refractory and Its Interaction with TiAl Alloy. *Materials* 2023, *16*, 7298

**DOI:** 10.3390/ma19081591

**Published:** 2026-04-15

**Authors:** Fuli Bian, Zheyu Cai, Jian Liu, Yu Liu, Man Zhang, Yixin Fu, Kailiang Zhu, Guangyao Chen, Chonghe Li

**Affiliations:** 1 Fire Research Institute of Shanghai of MEM, Shanghai 200032, China; bianfuli@shfri.cn (F.B.); zhukailiang@shfri.cn (K.Z.); 2 State Key Laboratory of Advanced Special Steel & Shanghai Key Laboratory of Advanced Ferrometallurgy & School of Materials Science and Engineering, Shanghai University, Shanghai 200444, China; zycai2022@shu.edu.cn (Z.C.); liuzuozuo@shu.edu.cn (J.L.); liuyucumt@shu.edu.cn (Y.L.); manzhang@shu.edu.cn (M.Z.); fyx13194382608@shu.edu.cn (Y.F.); 3 Shanghai Special Casting Engineering Technology Research Center, Shanghai 201605, China; 4 Zhejiang Institute of Advanced Materials, Shanghai University, Jiaxing 314100, China

Following publication, concerns were raised regarding the peer-review process related to the publication of this article [1]. Adhering to our standard procedure, the Editorial Board conducted an investigation, which determined that, while the peer-review process does comply with MDPI’s Editorial process policy (https://www.mdpi.com/editorial_process), the contribution of one of the four reviewers does not comply with MDPI’s Guideline for Reviewers (https://www.mdpi.com/reviewers#_bookmark11) or the expectations of the Editorial Board. As a result, the Editorial Board has decided to remove the contribution of Reviewer 4 from the open peer-review record (https://www.mdpi.com/1996-1944/16/23/7298/review_report). Following discussion with the authors, one reference originally included at the reviewer’s suggestion has also been removed from the article [1].

The reference [10] in the original publication, Cristescu, N.D.; Craciun, E.M.; Soós, E. *Mechanics of Elastic Composites*; Chapman and Hall/CRC: Boca Raton, FL, USA, 2004, has been removed. With this correction, the order of some references has been adjusted accordingly.

The authors state that the scientific conclusions are unaffected. This correction was approved by the Academic Editor. The original publication has also been updated.
